# INFUSION OF AGE-FRIENDLY PRINCIPLES INTO CURRICULA USING INTERACTIVE MODULES

**DOI:** 10.1093/geroni/igac059.129

**Published:** 2022-12-20

**Authors:** Sherry Greenberg, Shayle Adrian, Riad Twal

**Affiliations:** Monmouth University, Marjorie K. Unterberg School of Nursing & Health Studies, West Long Branch, New Jersey, United States; Seton Hall University, South Orange, New Jersey, United States; Seton Hall University, South Orange, New Jersey, United States

## Abstract

This presentation highlights the development of Age-Friendly care interactive modules to prepare the future healthcare workforce to care for older adults in Age-Friendly Health Systems. To date, there is a gap in the educational preparation of nurses, advanced practice nurses, and other health professionals to work in Age-Friendly Health Systems upon entry into practice. This project, funded by a faculty innovation grant at Seton Hall University, provides undergraduate and graduate nursing and interprofessional students with the background knowledge to care for older adults in Age-Friendly Health Systems. The aim of this project was to develop five interactive modules to embed in undergraduate and graduate nursing and interprofessional curricula focusing on the provision of care to older adults using the evidence-based 4Ms Framework: What Matters, Medication, Mentation, and Mobility. Faculty collaborated with instructional designers in development of the modules using Articulate 360 to embed them in curricula. The first module focuses on the background of the Age-Friendly Health Systems movement, how health systems may become an Age-Friendly Health System, and the evidence for the 4Ms Framework. The second module focuses on assessment and act on strategies related to What Matters to older adults including advance care planning strategies. The third module covers crucial information related to Medication such as avoiding potentially inappropriate medications, deprescribing, and antibiotic stewardship. The fourth module focuses on Mentation and covers assessment and act on strategies for depression, delirium, and dementia. The fifth module focuses on Mobility such as promoting mobility and decreasing fall risk.

